# Eye movements and postural control in dyslexic children performing different visual tasks

**DOI:** 10.1371/journal.pone.0198001

**Published:** 2018-05-24

**Authors:** Milena Razuk, José Angelo Barela, Hugo Peyre, Christophe Loic Gerard, Maria Pia Bucci

**Affiliations:** 1 UMR 1141 Inserm - Paris Diderot University, Robert Debré Hospital, Paris, France; 2 Institute of Physical Activity and Sport Sciences, Cruzeiro do Sul University, São Paulo, São Paulo, Brazil; 3 Institute of Biosciences, São Paulo State University, Rio Claro, São Paulo, Brazil; 4 Child and Adolescent Psychiatry Department, Robert Debré Hospital, Paris, France; Hangzhou Normal University, CHINA

## Abstract

The aim of this study was to examine eye movements and postural control performance among dyslexic children while reading a text and performing the Landolt reading task. Fifteen dyslexic and 15 non-dyslexic children were asked to stand upright while performing two experimental visual tasks: text reading and Landolt reading. In the text reading task, children were asked to silently read a text displayed on a monitor, while in the Landolt reading task, the letters in the text were replaced by closed circles and Landolt rings, and children were asked to scan each circle/ring in a reading-like fashion, from left to right, and to count the number of Landolt rings. Eye movements (Mobile T2^®^, SuriCog) and center of pressure excursions (Framiral^®^, Grasse, France) were recorded. Visual performance variables were total reading time, mean duration of fixation, number of pro- and retro-saccades, and amplitude of pro-saccades. Postural performance variable was the center of pressure area. The results showed that dyslexic children spent more time reading the text and had a longer duration of fixation than non-dyslexic children. However, no difference was observed between dyslexic and non-dyslexic children in the Landolt reading task. Dyslexic children performed a higher number of pro- and retro-saccades than non-dyslexic children in both text reading and Landolt reading tasks. Dyslexic children had smaller pro-saccade amplitude than non-dyslexic children in the text reading task. Finally, postural performance was poorer in dyslexic children than in non-dyslexic children. Reading difficulties in dyslexic children are related to eye movement strategies required to scan and obtain lexical and semantic meaning. However, postural control performance, which was poor in dyslexic children, is not related to lexical and semantic reading requirements and might not also be related to different eye movement behavior.

## Introduction

Dyslexic children do not perform sensory-motor tasks, including the postural control task [1–4] and eye movements task [5–8], in the same manner as non-dyslexic children. They usually perform poorly and demonstrate abnormal behavior in these tasks. Performance differences are even more pronounced when dyslexic children have to perform multiple tasks simultaneously, such as maintaining a stable posture while reading a text. Legrand and colleagues [9] observed that dyslexic children were more unstable while trying to maintain an upright stance than non-dyslexic children were, while silently reading a text. These results indicate that the attentional resources required for silent reading (a cognitive task) affect postural control functioning in dyslexic children. Similar results were observed when dyslexic children were asked to name simple objects (e.g., ball, table, and hat) [10].

Recently, researchers explored the effect of eye movements on postural control by recording eye movements (with an eye tracker) and postural stability simultaneously. For instance, Bucci and colleagues [4] explored the effect of fixation, pursuits, pro-saccades, and anti-saccades on postural control in dyslexic children and compared these results to those obtained from a group of non-dyslexic reading age-matched children and a group of non-dyslexic chronological age-matched children. The results showed that the quality of fixation and anti-saccade performance in dyslexic children was worse than that observed in non-dyslexic children [11]. Moreover, postural control performance was poorer in dyslexic children than in chronological age-matched non-dyslexic children. In addition, for all groups there was a reduction in postural sway values while performing saccades (pro- and retro-saccades) compared with fixation and pursuit tasks [11].

Bucci and colleagues [8] also compared eye movement recordings among dyslexic and non-dyslexic children in two different visual tasks, namely text reading and visual search, similar to the tasks used in the study by Prado et al. [12]. The visual search condition employed the same text used during the reading task, but the vowels were replaced with consonants and children were asked to count the occurrence of “r”s. Bucci and colleagues [8] observed atypical eye movement patterns (e.g., several fixations independent of the task and frequent backward saccades) in dyslexic children compared to non-dyslexic children. This finding is in line with previous findings [12], which suggested that changes in eye movements indicate that dyslexic children may have a reduced visual attentional (VA) window and this could equally affect reading as well as visual search during a reading task. In the aforementioned study, the VA window was defined as the number of distinct visual elements which can be processed [13] and according to the results, dyslexic children process less visual elements than non-dyslexic children do.

In order to examine the effects of cognition in reading tasks, non-lexical reading tasks, with spatial characteristics similar to that of the lexical task (i.e., number of letters, words and size of typefaces), were used. The Landolt reading task can be used for this purpose. In this task the visual structure of a written text is maintained but the letters are replaced by non-orthographic circle-like symbols, the so-called Landolt rings [14]. This seems to be a good strategy, which mimics the spatial characteristics of the required visual structure during reading without the additional influence of lexical, syntactic, or orthographic-phonological sources. To our knowledge, no study has examined eye movements in dyslexic and non-dyslexic children during the performance of the Landolt task.

In order to examine eye movements and postural performance in dyslexic children in the present study, we recorded eye movement during two different visual tasks (silent text reading and Landolt reading). Normal reading is a complex task that not only requires eye movements, but also perceptive and semantic processes to understand the words which are read. The Landolt reading task is important because by using it we can assess only eye movements during a reading task and avoid any cross effects of perceptive and semantic processes which might be different in dyslexic children, given their poor reading capabilities. Hillen and collaborators [14] showed that non-dyslexic young adults, performing the Landolt reading task, activated areas other than those related to semantic processing. Moreover, these authors also observed that eye movements were alike performing both reading a text and performing the Landolt reading task [14]. Therefore, the aim of the present study was to examine eye movements and postural control performance in dyslexic children while reading a text and performing the Landolt reading task. We hypothesized that dyslexic children would display different eye movements and poor postural control when reading a text compared to non-dyslexic children. On the other hand, eye movements and postural control performance would be similar between dyslexic and non-dyslexic children.

## Materials and methods

### Participants

Fifteen dyslexic children (age = 9.8±1.3 years, 2 girls and 13 boys) and fifteen IQ and age-matched non-dyslexic children (age = 10.0±1.3, 2 girls and 13 boys) participated in this study. Dyslexic children were recruited from the Child and Adolescent Psychiatry Department, Robert Debré Hopital (Paris, France) where they were referred for a complete evaluation of their dyslexia with an extensive examination, including neurological, psychological and phonological capabilities. Non-dyslexic children were recruited from the local community.

For each child, the time taken to read a text, understand the text, and the capacity to read words/pseudowords were evaluated using the L2MA battery [15]. This is a standard test developed by the Centre de Psychologie appliquée de Paris, often used in France and already employed in our previous studies, for selecting dyslexic population [11]. To be included in the study, children should have a score beyond 2 standard deviations and a normal mean intelligence quotient (IQ, evaluated with WISC-IV; between 80 and 115). All children underwent ophthalmologic/orthoptic examination for visual, sensorial, and motor function (mean values showed in Table 1). Visual acuity was normal (≥20/20) for all children. The stereoacuity threshold based on disparity detection was tested with the TNO random dot test for stereoscopic depth discrimination (Netherlands Organization, Richmond Products, Boca Raton, FL, USA) The near point of convergence (NPC) was normal for all children (mean value of 3 cm). Moreover, an evaluation of vergence fusion capability, using prisms and Maddox rod, was performed at near distance. The convergence and divergence amplitudes were significantly smaller in dyslexic children than in non-dyslexic children, with analysis of variance (ANOVA) showing significant effect of group for convergence and divergence (F_(1,28)_ = 29.863, p<0.01 and F_(1,28)_ = 13.211, p<0.01, respectively). In summary, orthoptic evaluation showed significant poor convergence and divergence amplitudes in dyslexic children.

**Table 1:** Clinical characteristics of all children (reading age-matched dyslexic and non-dyslexic children). Mean values of binocular vision (Stereoacuity test: TNO measured in seconds of arc; near point of convergence: NPC measured in cm, Vergence fusional amplitude (divergence and convergence) at near distance measured in prism diopters). Asterisks (*) indicate that value is significantly different compared to the group of dyslexic children (p<0.01).

Children group	TNO (sec of arc)	NPC (cm)	Convergence (pD)	Divergence (pD)
Non-dyslexic	60±1.7	3.6±0.4	38±3.6*	15.5±3.4*
Dyslexic	61±2.4	3.8±0.6	30.3±3.9	10.8±3.6

The investigation adhered to the principles of the Declaration of Helsinki and was approved by the Institutional Human Experimentation Committee (CPP Ile de France I, Hôpital Hotel-Dieu). Prior to enrolment in the experiment, written consent was obtained from parents of the children after an explanation of the experimental procedures.

### Visual tasks

All children performed two visual tasks: silent text reading and Landolt reading.

Silent text reading: A text of five lines was presented to each child on the screen of a computer. The mean character width was 0.5° and the text was written in black Courier font on a white background. The text was extracted from “Le géant égoïste” and “Monsieur Petit.” Each child was asked to read the text silently and after that the child had to narrate the story, ensuring that the reading task was well performed. All children were able to do so. Each child was asked to read normally.

Landolt reading: All letters in the text presented during the silent reading task were replaced with Landolt rings. The position of open Landolt rings was randomly distributed over the left, center, and right part of the entire stimulus in order to prevent the subjects from engaging in processing strategies. Each child was asked to scan each stimulus in a reading-like fashion from left to right and to identify the number of Landolt open rings observed.

A 22-inch computer monitor was used during both visual tasks, with a resolution of 1920 x 1080 pixels and refresh rate of 60 Hz.

#### Eye movement recording

Eye movements were recorded with the Mobile Eyebrain Tracker (Mobile EBT^®^, SuriCog), an eye-tracking device CE marked for medical purposes. The Mobile EBT^®^ uses cameras that capture the movements of each eye independently, with a typical precision of 0.25°. There is no obstruction of the visual field when using this recording system. Recording frequency was at 300 Hz.

#### Postural control recording

The excursions of the center of pressure (CoP) were recorded with Multitest Equilibre (Framiral^®^, Grasse, France) also called Balance Quest. The displacement of the CoP was sampled at 40 Hz and digitized with 16-bit precision. Postural recording was performed on a stable platform and occurred only in the first 30-second period of each visual reading task.

### Procedures

The experimental sessions took place in a dark room. Children were asked to stand upright, as still as possible, on the Framiral^®^ platform with parallel feet on footprints, with the arms along the side of the body (see for details Gouleme et al.,[16]). The computer monitor, on which the visual tasks were presented, was placed 60 cm away and adjusted to the eye level of each child. Each child was asked to read the text naturally (text reading task) and to identify the open character (the Landolt reading task). Each visual task was performed twice, making four trials in total, with the order of each trial defined randomly.

### Data analysis

Reading performance and eye movement analyses were performed using the MeyeAnalysis software, which automatically determined the onset and end of each saccade by using a built-in saccade detection algorithm. The onset and end of each saccade identified by the algorithm was visually inspected and verified by the investigator. Initially, the total reading time (s) was obtained. This corresponded to the elapsed time from the first/last eye movement used to initiate/finish the reading task. Based on each saccade detection, the following variables were obtained: duration of fixation (the total time (ms) between two successive saccades); number of pro- and retro-saccades; and amplitude of saccades (in degrees). Postural control performance was evaluated using the surface area of the CoP. Data from the CoP for each direction (anterior-posterior and medial-lateral) was obtained. The surface of the CoP, corresponding to the area of an ellipse encompassing 90% of all CoP data point excursions, was calculated. Because children performed two trials in each visual task, and all variables from the same condition was averaged for each child.

### Statistical analysis

After testing the normality and homogeneity of variance assumptions, repeated measures ANOVA with group (dyslexic and non-dyslexic) and visual tasks (text reading and Landolt reading), were performed for each dependent variable. When necessary, Tukey HSD post-hoc comparisons were performed as well. Analyses were performed using the SPSS software and the level of significance was kept at p < 0.05.

## Results

### Reading performance

**Fig 1:**
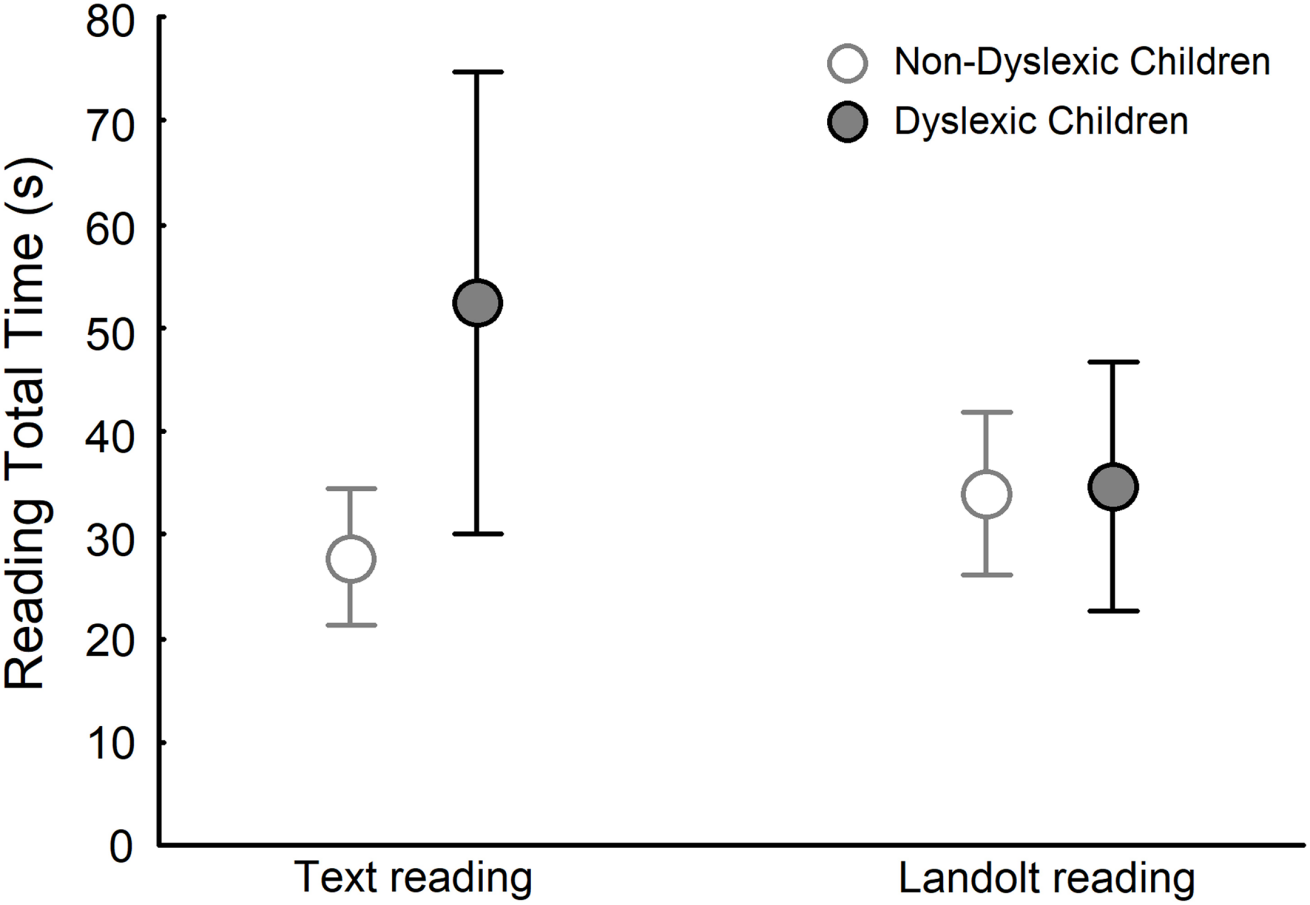
Total reading time for both non-dyslexic and dyslexic children during the text reading and Landolt reading tasks.

Fig 1 depicts the total reading time for dyslexic and non-dyslexic groups in both text reading and Landolt reading tasks. ANOVA showed a significant effect of group (F_(1,28)_ = 8.907, p<0.01), task (F_(1,28)_ = 4.981, p<0.05), and group and task interaction (F_(1,28)_ = 21.087, p<0.001). Post-hoc tests showed that in the text reading task, dyslexic children read slower than non-dyslexic children (q_(1,28)_ = 9.466, p<0.001). No difference was observed in the Landolt reading task between dyslexic and non-dyslexic children (q_(1,28)_ = 0.280, p>0.05). Finally, dyslexic children read faster in the Landolt reading task than in the text reading task (q_(1,28)_ = 6.824, p<0,001).

### Eye movement

**Fig 2:**
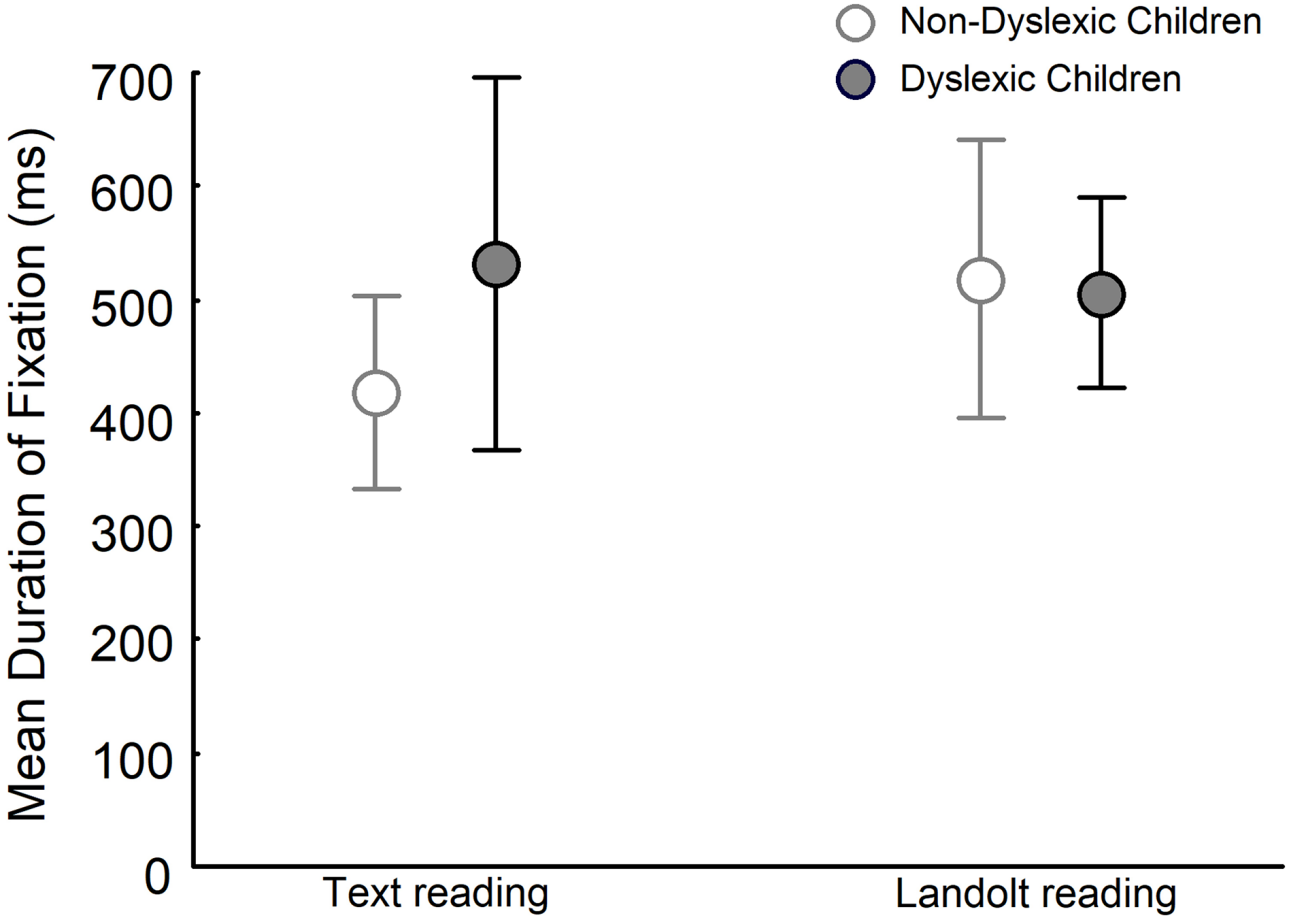
Mean duration of fixation for both non-dyslexic and dyslexic children in text reading and Landolt reading tasks.

Fig 2 depicts the mean duration of fixation for dyslexic and non-dyslexic groups in both text reading and Landolt reading tasks. ANOVA showed no significant effect of group (F_(1,28)_ = 2.030, p>0.05) and task (F_(1,28)_ = 2.208, p>0.05) but showed significant group and task interaction (F_(1,28)_ = 6.265, p<0.05). Post-hoc tests showed that in the text reading task, duration of fixation for dyslexic children was longer than that for non-dyslexic children (q_(1,28)_ = 4.530, p<0.03). No difference was observed in the Landolt reading task between dyslexic and non-dyslexic children (q_(1,28)_ = 0.477, p>0.05), but the duration of fixation for non-dyslexic children was longer during Landolt reading than that during text reading (q_(1,28)_ = 3.989, p<0.05). Duration of fixation was not different for dyslexic children in the Landolt reading and in the text reading tasks (q_(1,28_) = 1.011, p>0.05).

**Fig 3:**
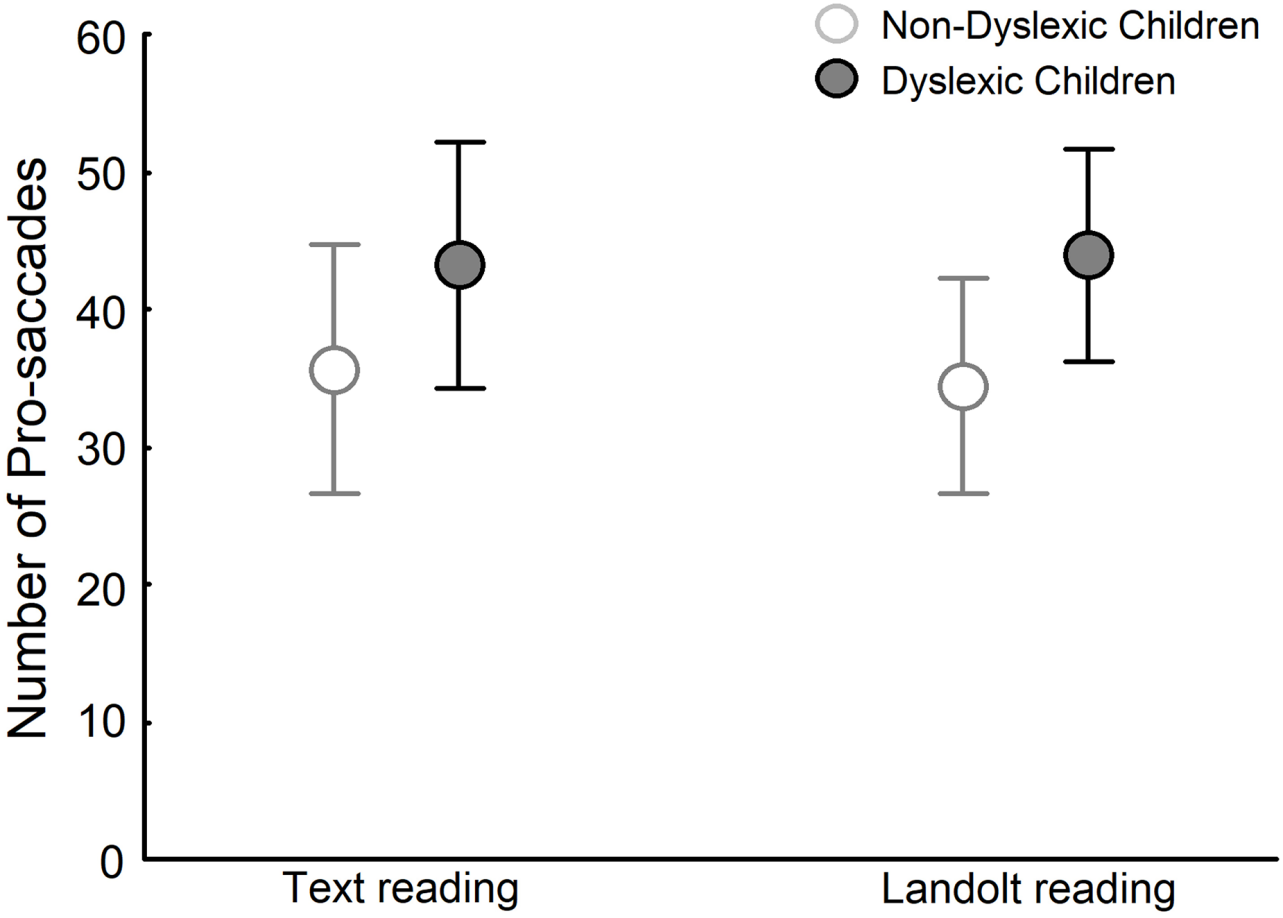
Number of pro-saccades for both non-dyslexic and dyslexic children in text reading and Landolt reading tasks.

Fig 3 depicts the number of pro-saccades for dyslexic and non-dyslexic groups in both text reading and Landolt reading tasks. ANOVA showed a significant effect of group (F_(1,28)_ = 11.079, p<0.01) but no effect of task (F_(1,28)_ = 0.029, p>0.05), and no group and task interaction (F_(1,28)_ = 0.321, p>0.05). Dyslexic children made more pro-saccades than did non-dyslexic children.

**Fig 4:**
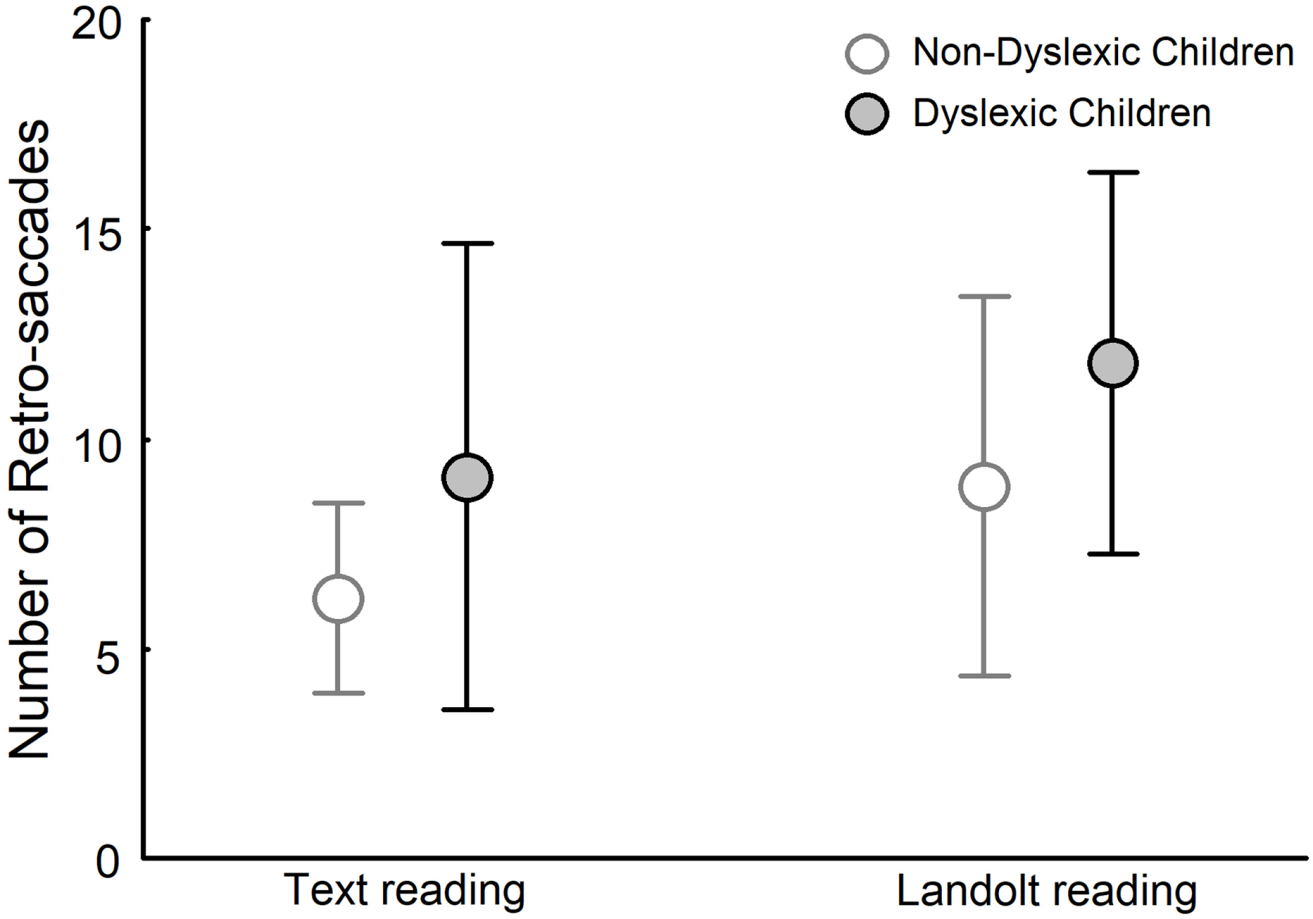
Number of retro-saccades for both non-dyslexic and dyslexic children in text reading and Landolt reading tasks.

Fig 4 depicts the number of retro-saccades for dyslexic and non-dyslexic groups in both text reading and Landolt reading tasks. ANOVA showed a significant effect of group (F_(1,28)_ = 4.977, p<0.05) and task (F_(1,28)_ = 12.289, p<0.001) but no effect of group and task interaction (F_(1,28)_ = 0.1853, p>0.05). The number of retro-saccades was higher in the Landolt reading task than in the text reading task for both dyslexic and non-dyslexic children. In addition, dyslexic children made more retro-saccades than did non-dyslexic children in both text reading and Landolt reading tasks.

**Fig 5:**
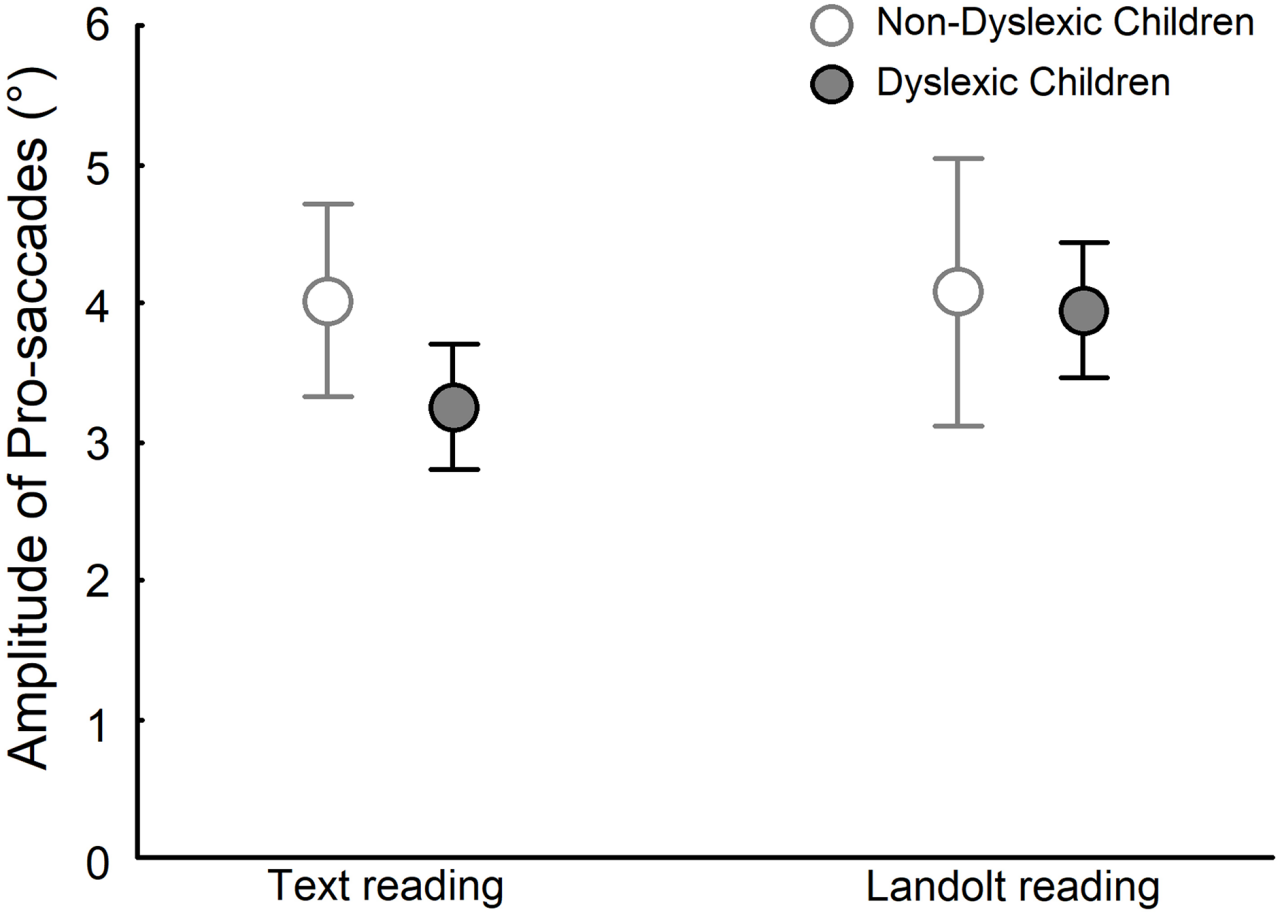
Amplitude of pro-saccades for both non-dyslexic and dyslexic children in text reading and Landolt reading tasks.

Fig 5 depicts the amplitude of pro-saccades for dyslexic and non-dyslexic groups in both text reading and Landolt reading tasks. ANOVA showed a significant effect of group (F_(1,28)_ = 4.708, p<0.05), task (F_(1,28)_ = 7.400, p<0.01), and group and task interaction (F_(1,28)_ = 5.035, p<0.05). Post-hoc tests showed that in the text reading task, the amplitude of pro-saccades in dyslexic children was smaller than that in non-dyslexic children (q_(1,28)_ = 5.434, p<0.004). No difference was observed in the Landolt reading task between dyslexic and non-dyslexic children (q_(1,28)_ = 0.947, p>0.05), but the amplitude of pro-saccades in dyslexic children was longer during Landolt reading task than in the text reading (q(1,28) = 4.962, p<0.01).

### Postural control

**Fig 6:**
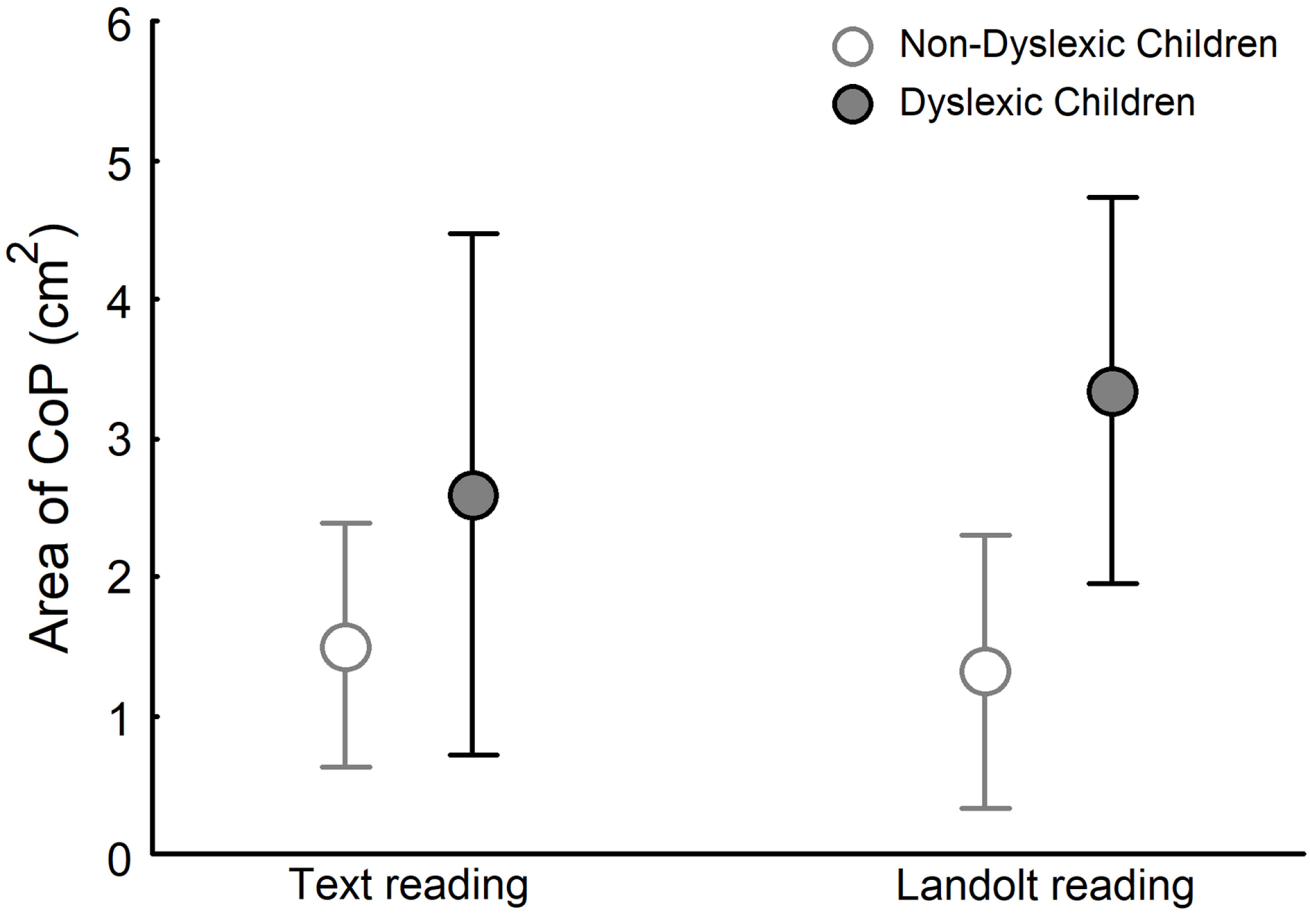
Area of CoP for both non-dyslexic and dyslexic children in text reading and Landolt reading tasks.

Fig 6 depicts the area of CoP for dyslexic and non-dyslexic groups in both text reading and Landolt reading tasks. ANOVA showed a significant effect of group (F_(1,28)_ = 11.802, p<0.01), no effect of task (F_(1,28)_ = 2.252, p>0.05), and significant group and task interaction (F_(1,28)_ = 6.255, p<0.01). Post-hoc tests showed that in the text reading task, the CoP area showed larger for dyslexic children than for non-dyslexic children (q_(1,28)_ = 5.861, p<0.001). In the Landolt reading task, CoP area for dyslexic children was larger than that for non-dyslexic children (q_(1,28)_ = 10.864, p<0.001). Moreover, post-hoc tests also showed that CoP area for dyslexic children in the Landolt reading task was larger than that for non-dyslexic children in the text reading task (q_(1,28)_ = 9.863, p<0.001).

## Discussion

The aim of this study was to examine eye movements and postural control performance of dyslexic children while reading a text and performing the Landolt reading task. Our results showed that dyslexic children need more time than non-dyslexic children to read text, but not during the performance of the Landolt reading task. Dyslexic children also employed different eye movement strategies during text reading, with longer fixation duration, more pro- and retro-saccades, and smaller amplitudes of saccades than non-dyslexic children. For the Landolt reading task, dyslexic children performed more pro-saccades and retro-saccades than did non-dyslexic children. Finally, dyslexic children performed worse than non-dyslexic children in maintaining an upright stance. These results and their implications are discussed below.

### Reading performance

Dyslexic children spent more time reading the text compared to non-dyslexic children. This result is not surprising and corroborates results from several other studies [12, 17–20], showing that dyslexia impairs the ability of children to read fluently. Despite demonstrating comprehension of the text content, dyslexic children need more time (almost twice) than non-dyslexic children in order to visually scan and decode the letters or syllable units.

Interestingly, the total reading time did not differ between dyslexic and non-dyslexic children in the Landolt reading task. Total reading time of dyslexic children, for the Landolt reading task, sharply decreased to the time observed for non-dyslexic children during the text reading task, which was not different from that observed for the Landolt reading task. These results are in line with findings from studies that employed visual search task similar to that required in the Landolt reading task [8, 12], where dyslexic children performed similarly to non-dyslexic children in the visual search task with consonant strings and visuospatial characteristics as seen in a reading task.

### Eye movement strategies

The longer time required to read a text, observed for dyslexic children, might be due to different eye movement strategies employed by dyslexic children. During text reading, the duration of fixation for dyslexic children was longer, and they also made more pro- and retro-saccades compared to non-dyslexic children. Moreover, the saccades were performed with smaller amplitudes. These results are similar to that of previous studies which observed longer fixation duration [8, 12], higher number of saccades [18, 19] and smaller saccade amplitudes [8, 18] in dyslexic children during reading, when compared to non-dyslexic children. Taking these results together, we can suggest that dyslexic children have longer fixation duration because they require a longer pause period in order to perform semantic processing [21]. Along with the fixation duration, dyslexic children also perform saccades with smaller amplitudes than non-dyslexic children. This might be related to difficulties in decoding letter- or syllable-units or number of letters/characters compared to non-dyslexic children [18, 22]. Finally, these two eye movement characteristics, longer fixation and smaller saccades amplitude, are also related to a higher number of saccades required for both advancing (pro-saccades) and retrieving (retro-saccades) the eye position in visual searching during reading.

During the Landolt reading task, however, no difference between dyslexic and non-dyslexic children was observed for the duration of fixation and amplitude of saccades. Interestingly, non-dyslexic children increased the duration of fixation, which indicates that they were not able to take advantage of the familiarity or automaticity of reading because most likely, they were not used to scanning the rings and closed circles required in the Landolt reading task. Dyslexic children showed similar fixation duration between text reading and Landolt reading tasks, suggesting that both tasks were performed without taking advantage of automaticity. This result corroborates previous observations when dyslexic children had to read a text and visually scan a set of letters, mimicking the Landolt reading task employed in this study [12]. These authors suggest that non-dyslexic children are able to increase/decrease the number of letters within a fixation, depending upon the familiarity of the task. In contrast, dyslexic children can only process a certain number of letters in each fixation, disregarding the task familiarity, and as a consequence the number of processed letters is similar in both reading and visual search [12]. This explanation may also apply to our results, with dyslexic children maintaining the same visual eye movement strategies but with more saccades and larger amplitudes.

Based on previous studies showing that the VA window of dyslexic children was reduced, our results suggest that dyslexic children can only process a few letters at each fixation and cannot increase the number of letters processed in a reading task as compared to visual search [8, 12]. Moreover, because there is no need for semantic processing in the Landolt reading task, dyslexic children can perform larger saccades, as observed, although still performing a higher number of pro- and retro-saccades.

### Postural control performance

Our results showed poor postural performance in dyslexic children compared to non-dyslexic children. This result is not surprising and corroborates results from several other studies [2, 3, 10, 11]. More interesting, however, is the fact that the postural performance of dyslexic children is also impaired in a non-familiar task, which is the Landolt reading task, employed in this study. A possible explanation for such larger sway during text reading and Landolt reading tasks would be that the visual search required becomes a secondary task affecting upright stance control. In this case, any visual search would impact the performance of posture control in dyslexic children. These results corroborate previous results showing deterioration of postural control performance when dyslexic children are required to maintain an upright stance and perform a secondary task simultaneously [10, 11].

The impact of performing the Landolt reading task on postural performance might question the possible cognitive impact of any lexical, syntactic, or orthographic-phonological reading on postural control in dyslexic children. Assuming that the Landolt paradigm minimizes such effects, the poor performance in maintaining an upright stance could most likely not be due to these issues. Poor postural control performance in dyslexic children has been observed in several visual conditions such as fixation and pursuit [11], visual manipulation [23], and even when vision cues are absent (eyes closed) [23]. Thus, deteriorating postural control performance in dyslexic children might be due to issues other than the lexical and semantic aspects of reading. Moreover, if this is the case, it might be that eye movement and postural control performance may not share a straight relationship. However, such suggestion still needs to be further examined.

In summary, reading difficulties in dyslexic children are not observed in visual tasks that do not require semantic and lexical processing, such as in the Landolt reading task. Improvement in performance in such a task is more likely due to different eye movement strategies. On the other hand, poor postural control performance in dyslexic children seems unrelated to any semantic or lexical requirement. Although with a few limitations, this study provides new and provocative pieces of information. The pioneer strategy in recording eye movements and postural performance simultaneously in children, both dyslexic and non-dyslexic, while reading a text and performing the Landolt reading task, seems to be important for uncovering several issues concerning the mechanisms underlying reading and postural control in dyslexic children.
